# Identification of minimal parameters for optimal suppression of chaos in dissipative driven systems

**DOI:** 10.1038/s41598-017-17969-9

**Published:** 2017-12-21

**Authors:** Pedro J. Martínez, Stefano Euzzor, Jason A. C. Gallas, Riccardo Meucci, Ricardo Chacón

**Affiliations:** 1 Departamento de Física Aplicada, E.I.N.A., Universidad de Zaragoza, E-50018 Zaragoza, Spain; 2 Instituto de Ciencia de Materiales de Aragón, CSIC-Universidad de Zaragoza, E-50009 Zaragoza, Spain; 3 Istituto Nazionale di Ottica, Consiglio Nazionale delle Ricerche, Largo E. Fermi 6, Firenze, Italy; 4 Departamento de Física, Universidade Federal da Paraíba, 58051-970 Joao Pessoa, Brazil; 5 Departamento de Física Aplicada, E.I.I., Universidad de Extremadura, Apartado Postal 382, E-06006 Badajoz, Spain; 6 Instituto de Computación Científica Avanzada (ICCAEx), Universidad de Extremadura, E-06006 Badajoz, Spain

**Keywords:** Electrical and electronic engineering, Computational science, Nonlinear phenomena

## Abstract

Taming chaos arising from dissipative non-autonomous nonlinear systems by applying additional harmonic excitations is a reliable and widely used procedure nowadays. But the suppressory effectiveness of generic non-harmonic periodic excitations continues to be a significant challenge both to our theoretical understanding and in practical applications. Here we show how the effectiveness of generic suppressory excitations is optimally enhanced when the impulse transmitted by them (time integral over two consecutive zeros) is judiciously controlled in a not obvious way. Specifically, the effective amplitude of the suppressory excitation is minimal when the impulse transmitted is maximum. Also, by lowering the impulse transmitted one obtains larger regularization areas in the initial phase difference-amplitude control plane, the price to be paid being the requirement of larger amplitudes. These two remarkable features, which constitute our definition of optimum control, are demonstrated experimentally by means of an analog version of a paradigmatic model, and confirmed numerically by simulations of such a damped driven system including the presence of noise. Our theoretical analysis shows that the controlling effect of varying the impulse is due to a subsequent variation of the energy transmitted by the suppressory excitation.

## Introduction

Obtaining full control of the chaotic dynamics of generic dissipative non-linear systems represents a fundamental interdisciplinary scientific and technological challenge. Among the different control procedures which have been proposed^1–3^, the application of judiciously chosen periodic excitations^4–20^ constitutes a reliable procedure in the context of dissipative non-autonomous systems. Hitherto, experimental control of chaos by periodic excitations has been demonstrated in many diverse systems, including laser systems^8,10,13,16^, neurological systems^11^, ferromagnetic systems^5^, chemical reactions^17^, and electronic systems^7,20^. It has been shown that the effectiveness of this non-feedback control procedure in non-autonomous systems depends critically upon the resonance condition and the initial phase difference between the primary (or chaos-inducing) periodic excitation and the secondary (or suppressory) periodic excitation, which has given rise to being called as phase control^19,20^. In such previous works, however, the flexibility of the control scenario against diversity in the suppressory excitations (SEs) was not studied since harmonic excitations have been overwhelmingly considered for the compelling reason of their simplicity. Clearly, the assumption of harmonic excitations means that the driving systems —whatever they might be —are effectively taken as linear. This mathematically convenient choice imposes a drastic and unnecessary restriction in the control scenario which is untenable for most natural and artificial systems due to their irreducible nonlinear nature^21^. Thus, to fully explore and exploit the physics of the control scenario, it seems appropriate to consider SEs exhibiting general features of periodic excitations which are the output of nonlinear systems, therefore being appropriately represented by Fourier series —not just by a single harmonic term—. It has been shown, in particular, that the suppressory effectiveness of periodic excitations seems to be highly sensitive to their wave forms^2^, while different types of wave forms have been considered in the contexts of impulsive control^22^ and time-delayed control^23^. Since there are infinitely many different waveforms, an important question, both scientifically and technologically, is how can one explain in physical terms —providing in turn a quantitative characterization —the effect of the SE’s waveform on the control scenario.

## Results

**Figure 1:**
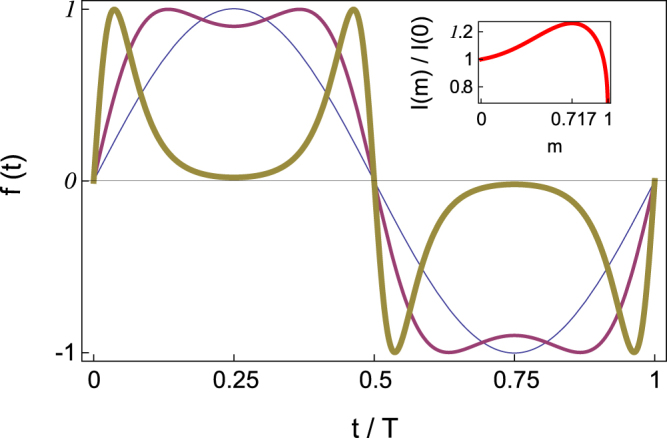
Suppressory *T*-periodic excitation *f*(*t*) versus *t/T* for three values of the shape parameter: *m* = 0 (sinusoidal pulse, thin line), $m=0.717\simeq \,{m}_{max}^{{\rm{i}}{\rm{m}}{\rm{p}}{\rm{u}}{\rm{l}}{\rm{s}}{\rm{e}}}$ (nearly square-wave pulse, regular line), and *m* = 0.9999 (double-humped pulse, thick line). The inset shows the corresponding normalized impulse *I*(*m*)/*I*(*m* = 0) versus *m*.

Here, we experimentally demonstrate that a relevant quantity properly characterizing the effectiveness of generic SEs *f*(*t*) having equidistant zeros in the control scenario is the *impulse* transmitted by the excitation over a half-period (hereafter referred to simply as the excitation’s impulse,

1 $$I\equiv {\int }_{0}^{T\mathrm{/2}}f(t)dt,$$

with *T* being the period)— a quantity integrating the conjoint effects of the excitation’s amplitude, period, and waveform. The relevance of the excitation’s impulse has been observed previously in such different contexts as adiabatically ac-driven periodic Hamiltonian systems^24^, chaotic dynamics of lasers^25^, and discrete soliton ratchets^26^, to cite just a few instances. For the sake of clarity, we consider an analog implementation of a simple paradigmatic model to discuss the impulse-induced chaos-control scenario: A damped-driven two-well Duffing oscillator described by the equation:

2 $$\ddot{x}=x-\beta [1+\eta \,f(t)]{x}^{3}-\delta \dot{x}+\gamma \,\cos \,(\omega t),$$

where all the variables and parameters are dimensionless (*β*, *η*, *δ*, *γ* > 0). The function *f*(*t*) is an unit-amplitude *T*-periodic excitation chosen to satisfy three remarkable properties. First, its waveform (and hence its impulse) is changed by solely varying a *single* parameter, the shape parameter *m*, between 0 and 1. Second, when *m* = 0, then $f{(t)}_{m=0}=\,\sin (2\pi t/T+\phi )$, with *φ* being the initial phase difference between the two excitations involved for all values of the shape parameter, i.e., one recovers the standard case^20^ of an harmonic excitation, while for the limiting value *m* = 1 the excitation and its impulse vanish. And third, as a function of *m*, the SE’s impulse presents a single maximum at a certain value $m={m}_{{\rm{\max }}}^{{\rm{impulse}}}$ (see Fig. 1 and the Supplemental Material^27^ for the definition and additional properties of *f*(*t*)). Here, *γ*cos(*ωt*) and −*βηx* ^3^ *f*(*t*) are to be regarded for convenience as the primary and suppressory excitations, respectively.

Also, we assume that, in the absence of any SE (*η* = 0), the Duffing oscillator (2) displays steady chaotic behavior which ultimately comes from a homoclinic bifurcation^28^, while we will focus here on the effective case of the main resonance (*T* = 2*π*/*ω*) between the two involved excitations in the presence of SEs (*η* > 0). As shown below, the simple and natural choice for *f*(*t*) allows us to characterize experimentally the genuine effect on the chaos-control scenario of the impulse transmitted by *generic* SEs, as well as to explain theoretically that the controlling effect of varying the impulse is due to a subsequent variation of the energy transmitted by the SE, allowing us to obtain useful analytical estimates of the chaotic threshold in the *φ* − *η* control plane from Melnikov^28^ and energy-based analyses, as is detailed in the Supplemental Material^27^.

**Figure 2:**
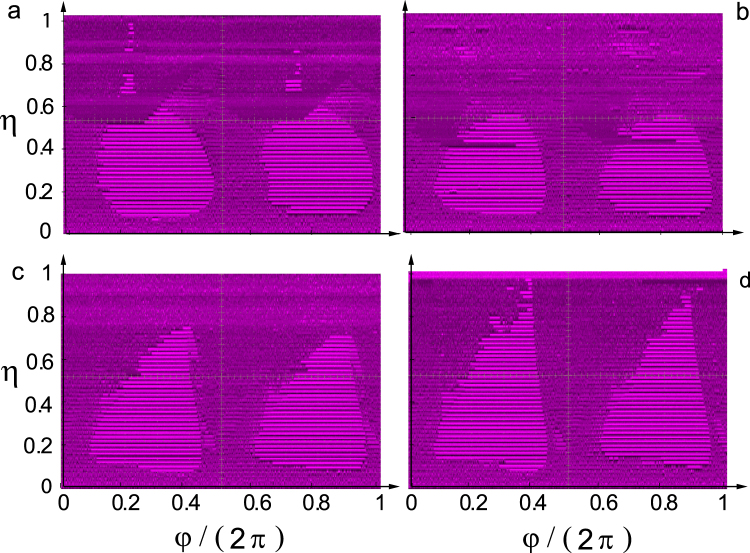
Experimentally obtained regions in the *φ* − *η* control plane with *φ* ∈ [0, 2*π*] and *η* ∈ [0, 1] corresponding to chaos (non-uniform magenta regions), low-energy periodic orbits around some of the two fixed points $(x=\pm {\beta }^{-\mathrm{1/2}},\mathop{x}\limits^{\mathrm{.}}=0)$ of the unperturbed Duffing oscillator (uniform light magenta regions), and higher-energy periodic orbits encircling both fixed points (uniform dark magenta regions) for four values of the shape parameter: (**a**) *m* = 0, (**b**) $m=0.717\simeq \,{m}_{max}^{impulse}$, (**c**) *m* = 0.9, and (**d**) *m* = 0.95. Fixed parameters: *δ* = 0.25, *γ* = 0.29, *β* = 1, *ω* = 1. We also observe black stripes separating consecutive uniform light magenta regions inside the two maximal islands of regularization centered at *φ*/(2*π*) = 0.25 and *φ*/(2*π*) = 0.75. These black stripes correspond to not visited zones of the phase space when the stabilized trajectories are around one of the two fixed points and scarcely observed outside of these islands.

We investigated the impulse-induced chaos-control scenario in the laboratory by implementing an analog version of the Duffing oscillator (2) (see^27^ for additional details). Our experimental results systematically indicate that complete regularization (i.e., periodic responses of any periodicity order) mainly appears inside two maximal islands in the *φ* − *η* control plane which are roughly symmetric with respect to the two optimal suppressory values ${\phi }_{opt}\equiv \{\pi \mathrm{/2,}\,3\pi \mathrm{/2}\}$, respectively, for all values of the shape parameter (see Fig. 2).

The analysis of the experimental data gives rise to the following genuine features of the present chaos-control scenario. While both the size and the shape of the boundaries of the maximal regularization islands vary as the SE’s impulse changes by solely varying *m*, they remain roughly centered around the optimal values ${\phi }_{opt}\equiv \{\pi \mathrm{/2,}\,3\pi \mathrm{/2}\}$ (note that the entire diagrams of Fig. 2 are periodic along the *φ*-axis, with fundamental period equal to *π*), confirming thus the theoretical predictions from Melnikov and energy-based analyses^27^.

**Figure 3:**
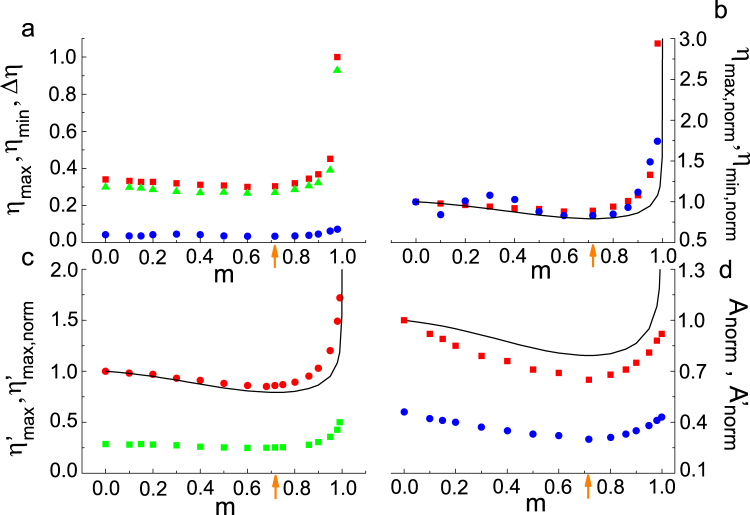
Experimental values of threshold amplitudes and regularization area in the control parameter plane versus shape parameter: (**a**) Lower threshold amplitude *η*_min_ (circles), upper threshold amplitude *η*_max_ (squares), and difference Δ*η* ≡ *η*_max_ − *η*_min_ (triangles) versus shape parameter *m*. (**b**) Normalized lower threshold amplitude ${\eta }_{\min ,norm}={\eta }_{{\rm{\min }},norm}(m)\equiv {\eta }_{{\rm{\min }}}(m)/{\eta }_{{\rm{\min }}}(m=\mathrm{0)}$ (circles), normalized upper threshold amplitude ${\eta }_{\max ,{norm}}={\eta }_{{\rm{\max }},norm}(m)\equiv {\eta }_{{\rm{\max }}}(m)/{\eta }_{{\rm{\max }}}(m=\mathrm{0)}$ (squares), and inverse of the normalized impulse [*I*(*m*)/*I*(*m* = 0)]^−1^ (solid line; cf. Eq. (S3) in Supplemental Material^27^). (**c**) Threshold amplitude ${\eta ^{\prime} }_{{\rm{\max }}}$ leading the Duffing oscillator to small-amplitude periodic oscillations around one of the fixed points $(x=\pm {\beta }^{-\mathrm{1/2}},\dot{x}=0)$ of the unperturbed Duffing oscillator (squares), its normalized version ${\eta ^{\prime} }_{\max ,norm}={\eta ^{\prime} }_{\max ,norm}(m)\equiv {\eta ^{\prime} }_{{\rm{\max }}}(m)/$ ${\eta ^{\prime} }_{{\rm{\max }}}(m=\mathrm{0)}$ (circles), and analytical estimate of the latter [solid line; cf. Eq. (3)]. (**d**) Normalized areas of regularized regions in the *φ* − *η* control plane, ${A}_{norm}={A}_{norm}(m)\equiv A(m)/A(m=0)$ (squares), ${A^{\prime} }_{norm}={A^{\prime} }_{norm}(m)\equiv A(m)/{A}_{total}$ (circles), in which *A*(*m*) and *A*_*total*_ are the regularization area and the total area, respectively. The solid line denotes the inverse of the normalized impulse [*I*(*m*)/*I*(*m* = 0)]^−1^, whereas the orange arrows indicate the value $m={m}_{max}^{impulse}\simeq \,0.717$, i.e., the *m* value at which the SE’s impulse is maximum. Fixed parameters: $\delta =0.25,\,\gamma =0.29,\,\beta =1,\,\omega =1$.

The lower, *η*_min_, and upper, *η*_max_, threshold values of the SE’s amplitude measured at the optimal suppressory values $\phi ={\phi }_{opt}\equiv \{\pi \mathrm{/2,}\,3\pi \mathrm{/2}\}$ as well as the difference $\Delta \eta \equiv {\eta }_{{\rm{\max }}}-{\eta }_{{\rm{\min }}}$ present, as functions of the shape parameter, a behavior quite similar to that of the inverse of the SE’ impulse [see Fig. 3(a)]. This can be seen more clearly in Fig. 3(b) in which it is shown the normalized amplitude thresholds ${\eta }_{{\rm{\max }}}(m)/{\eta }_{{\rm{\max }}}(m=\mathrm{0)}$, ${\eta }_{{\rm{\min }}}(m)/{\eta }_{{\rm{\min }}}(m=\mathrm{0)}$ together with the inverse of the normalized impulse [*I*(*m*)/*I*(*m* = 0)]^−1^ for the sake of comparison (see Supplemental Material^27^). In particular, we can see that the respective minima occur at values of the shape parameter which are very close in the sense that the difference between the corresponding values of the SE’s impulse is hardly noticeable.

Although we have not obtained a definitive explanation of the apparently anomalous behavior of *η*_min_ over a certain range of *small* values of *m*, it seems to be originated in the fractal character of the boundary for chaos in parameter space^29^ together with the fact that over such a range of *m* values the changes of the SE’s impulse are hardly noticeable^27^. The experimental results shown in Fig. 3(a) indicate that ever lower amplitudes *η*_min_ can suppress chaos as the impulse transmitted by the SE approaches its maximum value, whereas the corresponding suppressory ranges Δ*η* also decrease in the same way as *η*_min_ owing to the impulse-induced *enhancement* of the chaos-inducing effectiveness of the SE. This dependence of *η*_min,_ *η*_max_, Δ*η* on the SE’s impulse, which is theoretically anticipated from Melnikov analysis^27^, represents an essential feature of the present chaos-control scenario which is expected to be independent of the particular choice for the SE.

The lower values of the SE’s amplitude which suppress chaos and cause the Duffing oscillator to exhibit small-amplitude periodic oscillations around one of the fixed points $(x=\pm {\beta }^{-\mathrm{1/2}},\dot{x}=0)$ of the unperturbed Duffing oscillator (*δ* = *γ* = *η* = 0), ${\eta ^{\prime} }_{{\rm{\max }}}$, present, as a function of the shape parameter, a behavior quite similar to that of the inverse of the SE’s impulse [see Fig. 3(c)]. Remarkably, we can see in Fig. 3(c) that the theoretical estimate of its normalized version,

3 $$\frac{{\eta ^{\prime} }_{{\rm{\max }}}(m)}{{\eta ^{\prime} }_{{\rm{\max }}}(m=\mathrm{0)}}={[\frac{I(m)}{I(m=\mathrm{0)}}]}^{-1},$$

fits quite well the corresponding experimental values. Since the energy-based analysis giving rise to Eq. (3) is *general* in the sense that it can be applied to damped-driven systems of type (1) with generic (analytical) potentials *U*(*x*) (see Supplemental Material^27^), one may expect that the dependence of ${\eta ^{\prime} }_{{\rm{\max }}}$ on the SE’s impulse represents an additional generic feature of the present chaos-control scenario.

The total area of regularized regions (i.e., those associated with periodic responses of any periodicity order), *A*, in the *φ* − *η* control plane, presents, as a function of the shape parameter, a behavior which exhibits relevant features that are common to those of the inverse of the SE’s impulse. Specifically, Fig. 3(d) shows that its normalized versions ${A}_{norm}\equiv A(m)/A(m=0)$ and ${A^{\prime} }_{norm}\equiv A(m)/{A}_{total}$ present a single minimum just at $m={m}_{max}^{impulse}\simeq \,0.717$, i.e., the *m* value at which the SE’s impulse is maximum (see Fig. 1). It is worth noting that the same behavior is theoretically anticipated for the area of the aforementioned maximal islands from the application of the Melnikov analysis to the crudest approximation of the SE *f*(*t*), i.e., when solely the main harmonic of its Fourier expansion is retained (see Supplemental Material^27^ for an analytical estimate of the maximal islands’ area). This *inverse* dependence of the regularization areas in the *φ* − *η* control plane on the SE’s impulse represents an additional essential feature of the present chaos-control scenario which is expected to be especially useful in technological applications owing to it provides an useful criterion to guide the design of optimal SEs.

**Figure 4:**
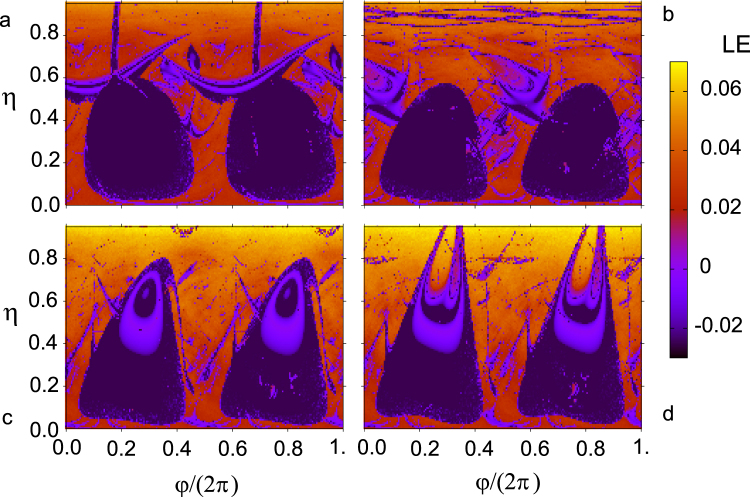
Numerically calculated maximal Lyapunov exponent in the *φ* − *η* control plane for four values of the shape parameter: (**a**) *m* = 0, (**b**) $m=0.717\simeq \,{m}_{max}^{impulse}$ (i.e., the *m* value at which the SE’s impulse is maximum), (**c**) *m* = 0.9 and (**d**) *m* = 0.95. Fixed parameters: *δ* = 0.25, *γ* = 0.29, *β* = 1, ω = 1.

Extensive computer simulations of Eq. (1) yielded numerical results from which we constructed three complementary types of diagrams providing useful information on both regularization regions in the *φ* − *η* control plane and the nature of the regularized (periodic) responses: maximal Lyapunov exponent, period-distribution, and isospike diagrams (see Supplemental Material^27^). The conclusions arising from the analysis of these diagrams systematically agree with all the aforementioned experimental features of the present chaos-control scenario, as can be appreciated by comparing the maximal Lyapunov exponent diagrams shown in Fig. 4 with the respective experimental diagrams shown in Fig. 3.

**Figure 5:**
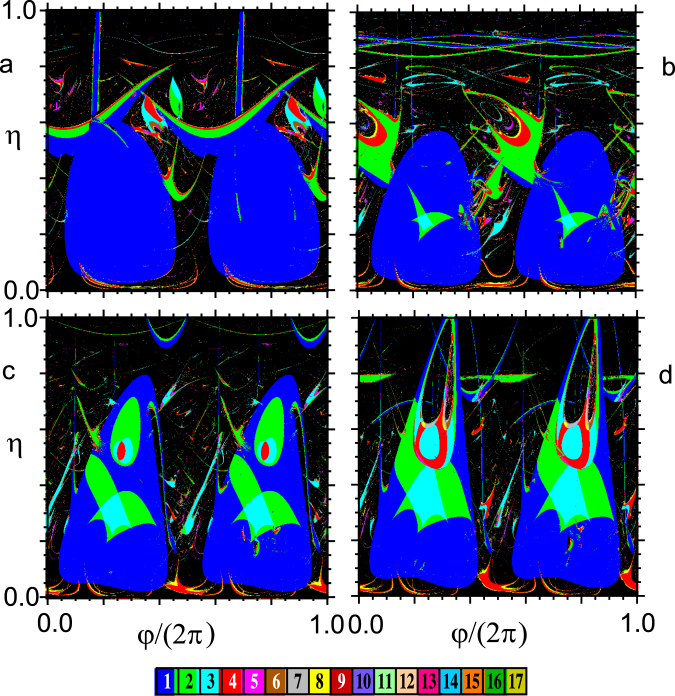
Numerically calculated regularization regions according to the waveform complexity (number of spikes or local maxima per period) of their solutions and chaotic regions (black) in the *φ* − *η* control plane for four values of the shape parameter: (**a**) *m* = 0, (**b**) $m=0.717\simeq \,{m}_{max}^{impulse}$ (i.e., the *m* value at which the SE’s impulse is maximum), (**c**) *m* = 0.9, and (**d**) *m* = 0.95. Fixed parameters: $\delta =\mathrm{0.25,}\,\gamma =\mathrm{0.29,}\,\beta =\mathrm{1,}\,\omega =1$.

Regarding the nature of the regularized responses, the period-distribution and isospike diagrams inform us of the existence of a wide spectrum of periodic responses in different regions of the *φ* − *η* control plane, the period-1 solutions being the predominant responses over the two maximal regularization islands irrespective of the values of the SE’s impulse (see Fig. 5 and Supplemental Material^27^).

Importantly, our numerical results show that the present chaos-control scenario is robust against the presence of moderate-intensity Gaussian noise, with the two maximal regularization islands being the most robust regularization regions, which represents an invaluable feature due to the unavoidable presence of thermal noise in many physical contexts, including for instance many nanoscale devices. Specific examples are shown in^27^.

## Methods

### Mathematical analysis

In the present work we consider the elliptic SE $f(t)\equiv N{\rm{sn}}(4Kt/T+{\rm{\Phi }}){\rm{dn}}(4Kt/T+{\rm{\Phi }})$, in which ${\rm{sn}}(\cdot )\equiv {\rm{sn}}(\cdot ;m)$ and ${\rm{dn}}(\cdot )\equiv {\rm{dn}}(\cdot ;m)$ are Jacobian elliptic functions of parameter *m* (*K* ≡ *K*(*m*) is the complete elliptic integral of the first kind)^32^, ${\rm{\Phi }}={\rm{\Phi }}(m,\phi )\equiv 2K(m)\phi /\pi$, *φ* ∈ [0, 2*π*], *T* ≡ 2*π*/*ω*, and $N=N(m)\equiv {[a+b{(1+\exp \{\frac{m-c}{d}\})}^{-1}]}^{-1}$, is a normalization function (*a* = 0.43932, *b* = 0.69796, *c* = 0.3727, *d* = 0.26883) which is introduced for the elliptic excitation to have the same amplitude, 1, and period *T*, for any waveform (i.e., $\forall m\in [\mathrm{0,}\,1]$). We applied the theory of elliptic functions^32^ to determine the properties of *f*(*t*). We applied Melnikov analysis^28^ to study the appearance and disappearance of chaos in parameter space.

#### Simulation

We used a Runge-Kutta fourth-order method to numerically study the purely deterministic case (2) as well as the robustness of the impulse-induced chaos-control scenario against the presence of additive noise in the Duffing equation: $\ddot{x}=x-\beta [1+\eta f(t)]{x}^{3}-\delta \mathop{x}\limits^{\mathrm{.}}+\gamma \,\cos (\omega t)+\sqrt{\sigma }\xi (t)$, where *ξ*(*t*) is a Gaussian white noise with zero mean and $\langle \xi (t)\xi (t+s)\rangle =\delta (s)$, and $\sigma =2{k}_{b}{T}^{\ast }$ with *k*_*b*_ and *T* ^*^ being the Boltzmann constant and temperature, respectively. We computed the Lyapunov exponents using a version of the algorithm introduced in^33^, with integration typically up to 10^4^ drive cycles for each fixed set of parameters.

#### Experiment

The experimental setup used in our analog implementation of the damped driven Duffing oscillator (2) is shown in Fig. S10 of the Supplemental Material^27^. The circuit is governed by the equation ${\zeta }^{-2}\ddot{x}=x-[1+\eta f(t)]{x}^{3}-{\zeta }^{-1}\delta \dot{x}+\gamma \,\cos (2\pi {f}_{d}t)$, $f(t)\equiv {a}_{0}(m)\sin (2\pi {f}_{c}t+\phi )+{a}_{1}(m)\sin (6\pi {f}_{c}t+3\phi )$, where $\zeta ={(RC)}^{-1}$ with *R* = 10 kΩ, *C* = 10 nF, while γ = 0.29 and *f*_*d*_ = 1592.500 Hz are the amplitude and frequency of the chaos-inducing signal, respectively, *δ* = 0.25, and *f*(*t*) is the two-harmonics approximation of the elliptic SE. After the transformation $t\to {\zeta }^{-1}t$, the circuit equation transforms into the dimensionless Eq. (2) with *ω* = 1. In the absence of any elliptic SE (*η* = 0), the circuit exhibits steady chaos for the above set of fixed parameters. The Duffing oscillator block with outputs *x* and *y* which is shown in Fig. S10 of the Supplemental Material^27^ has been detailed described in^34^. The initial phase difference *φ* has been implemented by selecting the frequency of the suppressory signal as *f*_*c*_ = *f*_*d*_ + 1/*T*_*sw*_ with *T*_*sw*_ being the sweeping phase period during which a phase variation of 2*π* occurs, with *T*_*sw*_ = 2 s in the experiments. The scan block generates two signals: a linear ramp *R*_*ϕ*_ for a phase variation of 2*π* and a 50 levels staircase signal *SC* (constant in amplitude during one phase sweep) allowing us to perform a sweeping of the suppressory amplitude *η*. The *x* and *y* signals from the Duffing oscillator block together with the phase-ramp and the *x* + *SC* signals are monitored on a four trace oscilloscope. Unlike the technique used in^20^, where a real-time automatic indicator was considered to discriminate between regular (periodic) and chaotic behaviour, we inspected here the temporal series of the *x* response signal for each point of the control-plane region *φ* ∈ [0, 2*π*], *η* ∈ [0, 1] according to the aforementioned resolution. This procedure provides us not only a reliable discrimination between chaotic and periodic responses but also to discriminate whether the periodic responses are low-energy orbits around some of the two fixed points $(x=\pm {\beta }^{-\mathrm{1/2}},\,\dot{x}=0)$ of the unperturbed Duffing oscillator (*δ* = *γ* = *η* = 0) or higher-energy orbits encircling both fixed points.

## Conclusions

During the last three decades or so^1–3^, and on the basis of an overwhelming use of harmonic SEs, the effectiveness of this particular type of SE has been systematically explored in a vast diversity of physical contexts by independently varying its amplitude and frequency as control parameters. However, by taking into account the irreducible nonlinear nature of real-world periodic excitations, the present results demonstrate that the SE’s impulse is the relevant quantity providing a complete characterization of the suppressory effectiveness of generic SEs by means of an exquisite control of the injection of energy into a chaotic damped-driven system. Specifically we have demostrated that the effective amplitude of the SE is minimal when the impulse transmitted is maximum. Also by lowering the SE’s impulse one obtains larger regularization areas in the *φ* − *η* control plane, the price to be paid being the requirement of larger values of the amplitude *η* while the optimal suppressory values *φ* = *φ*_*opt*_ ≡ {*π*/2, 3*π*/2} remain the same. These two new properties of the SE constitute our definition of optimum control. Future work may extend the present impulse-induced chaos-control scenario to the control of diverse quantum phenomena associated with the so-called quantum chaos, such as dynamical localization^30^ and quantum entanglement in systems in contact with environment^31^.

## Electronic supplementary material

Supplementary Information
