# Author Correction: Identification of minimal parameters for optimal suppression of chaos in dissipative driven systems

**DOI:** 10.1038/s41598-022-09255-0

**Published:** 2022-03-30

**Authors:** Pedro J. Martínez, Stefano Euzzor, Jason A. C. Gallas, Riccardo Meucci, Ricardo Chacón

**Affiliations:** 1 Departamento de Física Aplicada, E.I.N.A., Universidad de Zaragoza, E-50018 Zaragoza, Spain; 2 Instituto de Ciencia de Materiales de Aragón, CSIC-Universidad de Zaragoza, E-50009 Zaragoza, Spain; 3 Istituto Nazionale di Ottica, Consiglio Nazionale delle Ricerche, Largo E. Fermi 6, Firenze, Italy; 4 Departamento de Física, Universidade Federal da Paraíba, Joao Pessoa, 58051-970 Brazil; 5 Departamento de Física Aplicada, E.I.I., Universidad de Extremadura, Apartado Postal 382, E-06006 Badajoz, Spain; 6 Instituto de Computación Científica Avanzada (ICCAEx), Universidad de Extremadura, E-06006 Badajoz, Spain

Correction to: *Scientific Reports* 10.1038/s41598-017-17969-9, published online 21 December 2017

The original version of this Article contained an error in the Acknowledgments section.

"P.J.M. and R.C. acknowledge financial support from the Ministerio de Economía y Competitividad (MINECO, Spain) through FIS2011-25167 and FIS2012-34902 projects, respectively. R.C. acknowledges financial support from the Junta de Extremadura (JEx, Spain) through project GR15146. J.A.C.G. was supported by CNPq, Brazil."

now reads:

"P.J.M. and R.C. acknowledge financial support from the Ministerio de Economía y Competitividad (MINECO, Spain) through FIS2014-55867-P and FIS2011-25167 (P.J.M.) and FIS2012-34902 (R.C.) projects. R.C. acknowledges financial support from the Junta de Extremadura (JEx, Spain) through project GR15146. J.A.C.G. was supported by CNPq, Brazil."

The original Article has been corrected.

