# Supplementary material for: Identification of minimal parameters for optimal suppression of chaos in dissipative driven systems
Source: Sci Rep. 2017 Dec 21;7:17988. doi: 10.1038/s41598-017-17969-9 (PMC5740119; doi:10.1038/s41598-017-17969-9)
Supplement: Supplementary file 1 — Supplementary Information [file 41598_2017_17969_MOESM1_ESM.pdf]

Identification of minimal parameters for optimal suppression of chaos in dissipative driven systems

Pedro J. Martínez[1], Stefano Euzzor[2], Jason A. C. Gallas[3], Riccardo Meucci[2] and Ricardo Chacón[4]

[1]*Departamento de Física Aplicada, E.I.N.A.,*
Universidad de Zaragoza, E-50018 Zaragoza, Spain,
and Instituto de Ciencia de Materiales de Aragón,
CSIC-Universidad de Zaragoza, E-50009 Zaragoza, Spain

[2]*Istituto Nazionale di Ottica, Consiglio Nazionale delle Ricerche, Largo E. Fermi 6, Firenze, Italy*

[3]*Departamento de Física, Universidade Federal da Paraíba, 58051-970 Joao Pessoa, Brazil and*

[4]*Departamento de Física Aplicada, E.I.I., Universidad de Extremadura,*
Apartado Postal 382, E-06006 Badajoz, Spain,
and Instituto de Computación Científica Avanzada (ICCAEx),
Universidad de Extremadura, E-06006 Badajoz, Spain

SUPPLEMENTARY INFORMATION

I. THEORETICAL METHODS

A. Fourier expansion of the suppressory excitation (SE)

In our study we consider the elliptic SE

$$f(t) \equiv N \operatorname{sn}(4Kt/T + \Phi) \operatorname{dn}(4Kt/T + \Phi), \tag{S1}$$

in which $\operatorname{sn}(\cdot) \equiv \operatorname{sn}(\cdot; m)$ and $\operatorname{dn}(\cdot) \equiv \operatorname{dn}(\cdot; m)$ are Jacobian elliptic functions of parameter m ($K \equiv K(m)$ is the complete elliptic integral of the first kind) [1], $\Phi = \Phi(m, \varphi) \equiv 2K(m)\varphi/\pi$, $\varphi \in [0, 2\pi]$, $T \equiv 2\pi/\omega$, and

$$N = N(m) \equiv \left[a + b \left(1 + \exp\left\{ \frac{m-c}{d} \right\} \right)^{-1} \right]^{-1}, \tag{S2}$$

is a normalization function ($a \equiv 0.43932, b \equiv 0.69796, c \equiv 0.3727, d \equiv 0.26883$) which is introduced for the elliptic excitation to have the same amplitude, 1, and period T, for any waveform (i.e., $\forall m \in [0, 1]$). When $m = 0$, then $f(t)_{m=0} = \sin(2\pi t/T + \varphi)$, i.e., one recovers the standard case of an harmonic SE, while for the limiting value $m = 1$ the excitation vanishes. The effect of

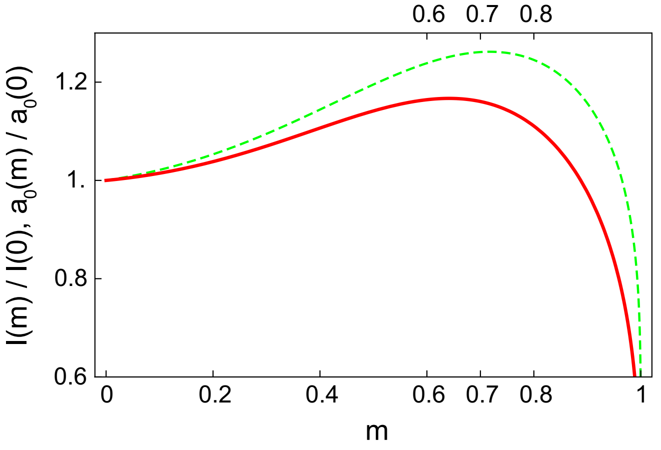

Figure S1: **Comparison between the SE's impulse and its first Fourier coefficient as functions of the shape parameter.** Normalized first Fourier coefficient $a_0(m)/a_0(m=0)$ (Eq. S5, solid line) and SE's impulse $I(m)/I(m=0) \equiv \pi N(m)/(2K(m))$ (Eq. S3, dashed line) versus shape parameter m. We can see that the respective single maxima occur at very close values of the shape parameter: $m_{\max}(n=0) \simeq 0.642$ and $m_{\max}^{impulse} \simeq 0.717$, respectively.

renormalization of the elliptic arguments is clear: with T constant, solely the excitation's impulse is varied by increasing the shape parameter m from 0 to 1. Note that, as a function of m, the SE's impulse per unit of amplitude and unit of period

$$I = I(m) \equiv \frac{N(m)}{2K(m)} \tag{S3}$$

presents a single maximum at $m = m_{\max}^{impulse} \simeq 0.717$ (see Fig. 1 of the main text).

The Fourier expansion of the elliptic SE (Eq. S1) reads

$$f(t) = \sum_{n=0}^{\infty} a_n(m) \sin\left[(2n+1)\left(\frac{2\pi t}{T} + \varphi\right)\right], \tag{S4}$$

$$a_n(m) \equiv \frac{\pi^2 N(m)(n+\frac{1}{2})}{\sqrt{m}K^2(m)} \operatorname{sech}\left[\frac{(n+\frac{1}{2})\pi K(1-m)}{K(m)}\right], \tag{S5}$$

in which its Fourier coefficients satisfy the properties: i) $\lim_{m\to 1} a_n(m) = 0$, ii) $a_n(m)$ exhibits a single maximum at $m = m_{\max}(n)$ such that $m_{\max}(n+1) > m_{\max}(n)$, $n = 0, 1, ...$, iii) the normalized functions $a_0(m)/a_0(m=0)$ and $I(m,T)/I(m=0,T) \equiv \pi N(m)/(2K(m))$ present, as functions of m, similar behaviours while their maxima verify that $m_{\max}(n=0) \simeq 0.65$ is very close to $m_{\max}^{impulse} \simeq 0.717$ (see Fig. S1), and iv) the Fourier expansion (Eq. S4) is rapidly convergent over a wide range of values of the shape parameter. The following remarks may now be in order.

First, regarding analytical estimates, the property (iii) is relevant in the sense that it allows us to obtain an useful effective estimate of the chaotic threshold in the $\varphi - \eta$ control plane from

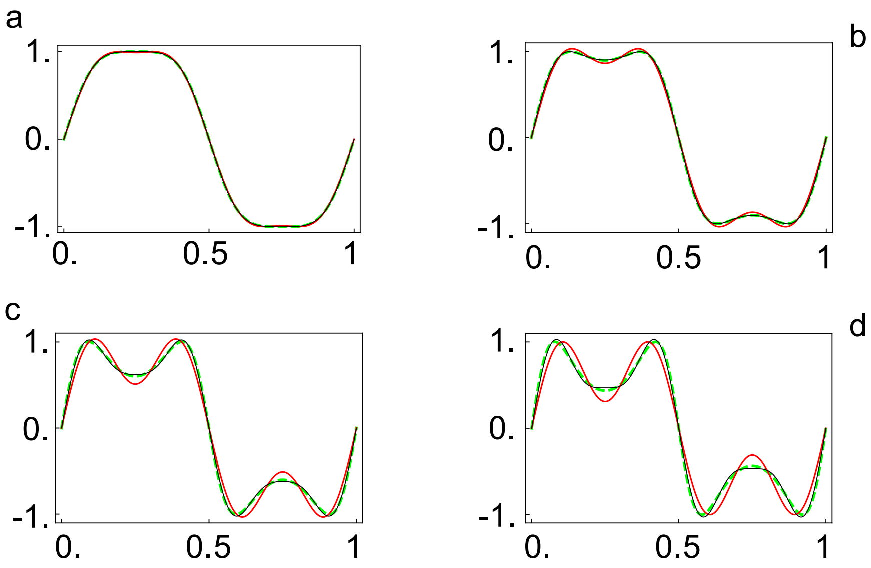

Figure S2: **Comparison between the elliptic SE and its two- and three-harmonics approximations over a period for four values of the shape parameter.** Plots of the elliptic SE (Eq. S1, dashed line), its two-harmonics approximation $S_2(t) \equiv a_0(m)\sin(\omega t+\varphi)+a_1(m)\sin(3\omega t+3\varphi)$ (cf. Eqs. S4 and S5, solid line), and its three-harmonics approximation $S_3(t) \equiv a_0(m)\sin(\omega t+\varphi)+a_1(m)\sin(3\omega t+3\varphi)+a_2(m)\sin(5\omega t+5\varphi)$ (cf. Eqs. S4 and S5, thin solid line) versus time for four values of the shape parameter: **a**, $m=0.5$; **b**, $m=0.717\simeq m_{\max}^{impulse}$; **c**, $m=0.9$; **d**, $m=0.95$.

Melnikov analysis (MA) [2,3] by solely retaining the first harmonic of the Fourier expansion (Eq. S4):

$$f(t)\approx a_0(m)\sin(\omega t+\varphi)\,. \tag{S6}$$

Second, regarding experiments, the property (iv) is relevant in the sense that it allows us to effectively approximate the elliptic SE by solely retaining the first two harmonics of its Fourier expansion over the range of values of the shape parameter of our interest ($0\leqslant m\lesssim 0.95$; see Fig. S2):

$$f(t;T,m,\varphi)\approx a_0(m)\sin(\omega t+\varphi)+a_1(m)\sin(3\omega t+3\varphi)\,. \tag{S7}$$

Third, regarding numerical simulations, we considered the entire Fourier expansion of the elliptic SE in order to obtain useful information concerning the effectiveness of the approximations used in the theoretical analysis and experiments (cf. Eqs. S6 and S7, respectively).

B. Chaotic threshold from Melnikov analysis

Melnikov introduced a function (now known as the Melnikov function (MF), $M(t_0)$) which measures the distance between the perturbed stable and unstable manifolds in the Poincaré section at t_0. If the MF presents a simple zero, the manifolds intersect transversally and chaotic instabilities result. See Refs. [2,3] for more details about MA. Regarding Eq. (2) in the main text, note that keeping with the assumption of the MA, it is assumed that one can write $\delta = \varepsilon\bar{\delta}, \gamma = \varepsilon\overline{\gamma}, \eta = \varepsilon\bar{\eta}$ where $0 < \varepsilon \ll 1$ while $\bar{\delta}, \overline{\gamma}, \bar{\eta}, \beta, \omega$ are of order one. Thus, the application of MA to Eq. (2) in the main text yields the MF

$$M^{\pm}(t_0) = -D \pm A\sin(\omega t_0) + \frac{\pi\eta}{6\beta}\sum_{p=0}^{\infty} a_p(m)\, b_p(T)\cos\left[\Omega_p(T)\, t_0 + (2p+1)\varphi\right], \tag{S8}$$

$$D \equiv \frac{4\delta}{3\beta}, \tag{S9}$$

$$A \equiv \sqrt{\frac{2}{\beta}}\pi\gamma\omega\,\mathrm{sech}(\pi\omega/2), \tag{S10}$$

$$\Omega_p(T) \equiv (2p+1)\frac{2\pi}{T}, \tag{S11}$$

$$b_p(T) \equiv \Omega_p^2\left(4+\Omega_p^2\right)\mathrm{csch}\left(\frac{\pi\Omega_p}{2}\right), \tag{S12}$$

where the coefficients $a_p(m)$ are given by Eq. S5, and where the positive (negative) sign refers to the right (left) homoclinic orbit of the underlying conservative Duffing oscillator ($\delta = \eta = \gamma = 0$):

$$x_{0,\pm}(t) = \pm\sqrt{\frac{2}{\beta}}\,\mathrm{sech}(t), \tag{S13}$$

$$\dot{x}_{0,\pm}(t) = \mp\sqrt{\frac{2}{\beta}}\,\mathrm{sech}(t)\tanh(t). \tag{S14}$$

Let us assume that, in the absence of any SE ($\eta = 0$), the damped driven two-well Duffing oscillator (Eq. 2 in the main text) presents chaotic behaviour for which the respective MF,

$$M_0^{\pm}(t_0) \equiv -D \pm A\sin(\omega t_0), \tag{S15}$$

has simple zeros, i.e., $D \leqslant A$ or

$$\gamma \geqslant \gamma_{th} \equiv \frac{2\sqrt{2}\delta\cosh(\pi\omega/2)}{3\pi\sqrt{\beta}\omega}, \tag{S16}$$

where the equal sign corresponds to the case of tangency between the stable and unstable manifolds [3]. If we now let the SE act on the Duffing oscillator such that $B^* \leqslant A - D$, with

$$B^* \equiv \max_{t_0}\left\{\frac{\pi\eta}{6\beta}\sum_{p=0}^{\infty} a_p(m)\, b_p(T)\cos\left[\Omega_p t_0 + (2p+1)\varphi\right]\right\}, \tag{S17}$$

then this relationship represents a sufficient condition for $M^{\pm}(t_0)$ to change sign at some t_0. Thus, a necessary condition for $M^{\pm}(t_0)$ to always have the same sign is

$$B^* > A - D \equiv B_{\min}. \tag{S18}$$

Since $a_p(m) > 0, b_p(T) > 0, p = 0, 1, 2, ...$, one straightforwardly obtains

$$B^* \leqslant \frac{\pi\eta}{6\beta} \sum_{p=0}^{\infty} a_p(m)\, b_p(T), \tag{S19}$$

and hence,

$$\eta > \eta_{\min} \equiv \left(1 - \frac{D}{A}\right) R, \tag{S20}$$

$$R \equiv \frac{6\beta A}{\pi \sum_{p=0}^{\infty} a_p(m)\, b_p(T)}. \tag{S21}$$

Note that Eq. S20 provides a lower threshold for the amplitude of the SE. Similarly, an upper threshold is obtained by imposing the condition that the SE may not enhance the initial chaotic state (i.e., it does not increase the (initial) gap from the homoclinic tangency condition),

$$B^* \leqslant \frac{\pi\eta}{6\beta} \sum_{p=0}^{\infty} a_p(m)\, b_p(T) < A + D \equiv B_{\max}, \tag{S22}$$

and hence,

$$\eta < \eta_{\max} \equiv \left(1 + \frac{D}{A}\right) R, \tag{S23}$$

which is a necessary condition for $M^{\pm}(t_0)$ to always have the same sign. Thus, the suitable (suppressory) amplitudes of the SE must satisfy

$$\eta_{\min} < \eta < \eta_{\max}, \tag{S24}$$

while the width of the range of suitable amplitudes reads

$$\Delta\eta \equiv \eta_{\max} - \eta_{\min} = \frac{16\delta}{\pi \sum_{p=0}^{\infty} a_p(m)\, b_p(T)}. \tag{S25}$$

Figures S3 and S4 show how both the width of the range of suitable amplitudes $\Delta\eta$ (Eq. S25) and the threshold amplitudes $\eta_{\min}, \eta_{\max}$ present a single minimum at $m = m_{\min}$ as the shape parameter m is increased from 0 to 1 due to the dependence of the function R on the shape parameter. While this minimum $m_{\min} \equiv m_{\min}(T)$ is very near $m_{\max}^{impulse} \simeq 0.717$ over a wide range of periods, one cannot expect an exact agreement between $m_{\min}$ and $m_{\max}^{impulse}$ for all periods owing to the dependence of the chaotic threshold on the common excitation period (main resonance).

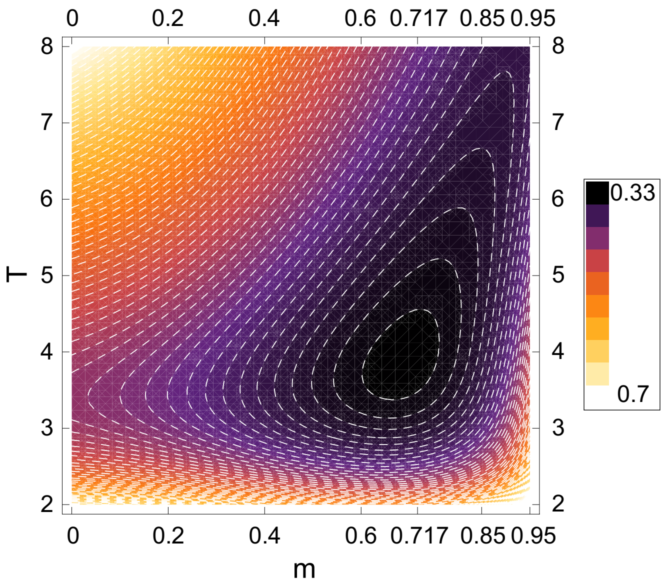

Figure S3: **Width of the range of suitable suppressory amplitudes in the $m - T$ control plane.** Contour plot of the function $\Delta\eta \equiv \eta_{\max} - \eta_{\min}$ (Eq. S24) versus shape parameter m and period T. Note the existence of an absolute minimum at $m \simeq m_{\max}^{impulse}, T \simeq 4$. System parameters: $\gamma = 0.29, \delta = 0.25, \beta = 1$.

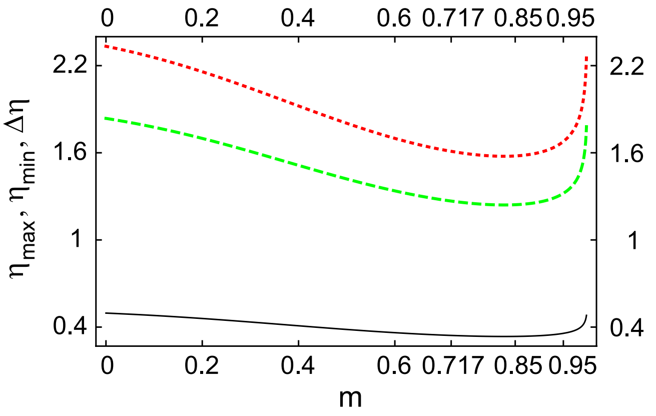

Figure S4: **Threshold amplitudes and width of the range of suitable suppressory amplitudes versus shape parameter.** Upper threshold amplitude $\eta_{\max}$ (Eq. S22, dotted line), lower threshold amplitude $\eta_{\min}$ (Eq. S19, solid line), and difference $\Delta\eta \equiv \eta_{\max} - \eta_{\min}$ (Eq. S24, dashed line) versus shape parameter m. $\omega = 1$ and the remaining parameters as in Fig. S3.

This means that ever lower amplitudes $\eta_{\min}$ can suppress chaos as the impulse transmitted by the SE approaches its maximum value, whereas the corresponding suppressory ranges $\Delta\eta$ also decrease in the *same* way as $\eta_{\min}$ owing to the impulse-induced enhancement of the chaos-inducing effectiveness of the SE. This dependence of $\eta_{\max}, \eta_{\min}, \Delta\eta$ on the SE's impulse represents a genuine feature of the impulse-induced chaos-control scenario.

Regarding suitable values of the initial phase difference φ, note that φ determines the relative phase between $M_0^{\pm}(t_0)$ and

$$\frac{\pi\eta}{6\beta}\sum_{p=0}^{\infty}a_p(m)\,b_p(T)\cos\left[\Omega_p t_0+(2p+1)\,\varphi\right]$$

irrespective of the shape parameter value. We, therefore, conclude from previous theory [4] that a sufficient condition for $\eta_{\min}<\eta<\eta_{\max}$ to also be a sufficient condition for suppressing chaos is that $M_0^{\pm}(t_0)$ and

$$\frac{\pi\eta_{\min,\max}}{6\beta}\sum_{p=0}^{\infty}a_p(m)\,b_p(T)\cos\left[\Omega_p t_0+(2p+1)\,\varphi\right]$$

are in opposition. This yields the optimum suppressory values

$$\varphi_{opt}\equiv\left\{\frac{\pi}{2},\frac{3\pi}{2}\right\} \tag{S26}$$

for *all* $m\in[0,1]$ in the sense that they allow the widest amplitude ranges for the elliptic SE.

To obtain an useful analytical estimate of the boundaries of the regions in the $\varphi-\eta$ control plane where chaos is suppressed, we assume the first-harmonic approximation given by Eq. S6 instead of the entire Fourier expansion (cf. Eq. S4) in the remainder of this section. Indeed, recall that the value $m_{\max}^{impulse}\simeq 0.717$ at which the SE's impulse presents a single maximum is very close to the value $m=m_{\max}(n=0)\simeq 0.642$ where the amplitude $a_0(m)$ (cf. Eq. S5) presents a single maximum (see Fig. S1). Thus, we apply MA to the *effective* MF

$$M_{eff}^{\pm}(t_0)=-D\pm A\sin(\omega t_0)+B_0\cos(\omega t_0+\varphi)\,, \tag{S27}$$

$$B_0\equiv\frac{\pi\eta}{6\beta}a_0(m)\,b_0(T)\,, \tag{S28}$$

while the effectiveness of the first-harmonic approximation ($\eta>0$) at suppressing chaos will be examined by considering for example the effective MF $M_{eff}^{+}(t_0)$ (the analysis of $M_{eff}^{-}(t_0)$ is similar and leads to the same conclusions). To this end, it is convenient to use the normalized MF $M_n^{+}(t_0)\equiv M_{eff}^{+}(t_0)/D$ to write

$$\begin{aligned}M_n^{+}(t_0)&=-1+\left(R'-R''\sin\varphi\right)\sin(\omega t_0)+R''\cos\varphi\cos(\omega t_0)\\&\leqslant-1+\sqrt{\left(R'-R''\sin\varphi\right)^2+R''^2\cos^2\varphi},\end{aligned} \tag{S29}$$

where $R'\equiv A/D, R''\equiv B_0/D$. If one now lets the first-harmonic approximation act on the system such that

$$\left(R'-R''\sin\varphi\right)^2+R''^2\cos^2\varphi\leqslant 1, \tag{S30}$$

this relationship represents a sufficient condition for $M_n^+(t_0)$ to be negative (or null) for all t_0. The equals sign in Eq. S30 yields the boundary of the region in the $\varphi-\eta$ plane where chaos is suppressed:

$$\eta=\frac{6\sqrt{2\beta}\gamma\tanh\left(\pi\omega/2\right)}{a_0\left(m\right)\omega\left(4+\omega^2\right)}\left[\sin\varphi\pm\sqrt{\frac{\gamma_{th}^2}{\gamma^2}-\cos^2\varphi}\right], \tag{S31}$$

with $\gamma>\gamma_{th}$ (cf. Eq. S16), and where the two signs before the square root correspond to the two branches of the boundary (see Fig. S5). The following remarks may now be in order.

First, the boundary function (Eq. S31) represents two loops encircling the regularization regions in the $\varphi-\eta$ plane which are symmetric with respect to the optimal suppressory values

$$\varphi_{opt}\equiv\left\{\frac{\pi}{2},\frac{3\pi}{2}\right\}, \tag{S32}$$

respectively, i.e., those values of the initial phase difference for which the range of suitable suppressory values of η is maximum. As expected, they are the same suppressory values than those found in the exact case of representing the elliptic SE by its entire Fourier expansion (cf. Eq. S26).

Second, the area, A_R, enclosed by the boundary function (Eq. S31) is straightforwardly obtained from previous theory [4]:

$$A_R=\frac{32\delta\sinh\left(\pi\omega/2\right)}{\pi a_0(m)\left(4\omega^2+\omega^4\right)}. \tag{S33}$$

Observe that one finds $A_R\rightarrow 0$ as $\delta\rightarrow 0$, which corresponds to the limiting Hamiltonian case, as expected. More importantly, the normalized regularization area

$$\frac{A_R(m)}{A_R(m=0)}=\frac{a_0(m=0)}{a_0(m)} \tag{S34}$$

presents, as a function of the shape parameter, a single minimum at the m value where $a_0(m)$ presents a single maximum (see Fig. S1): $m_{\max}\left(n=0\right)\simeq 0.642$, which is very close to $m_{\max}^{impulse}\simeq 0.717$. This *inverse* dependence of the regularization area on the SE's impulse represents a genuine feature of the impulse-induced chaos-control scenario.

Third, the regularization area shrinks as the ratio γ_{th}/γ diminishes, which means that the impulse-induced chaos-control scenario is *sensitive* to the strength of the initial chaotic state in the sense of its proximity to the threshold condition (cf. Eq. S16).

C. Energy-based analysis

By analyzing the variation of the Duffing oscillator's energy, one straightforwardly obtains an alternative physical explanation of the foregoing MA-based predictions. Indeed, Eq. 2 in the main

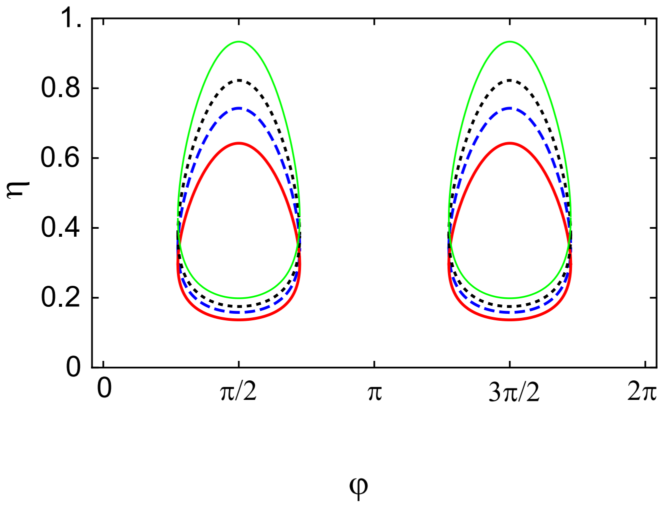

Figure S5: **Analytical estimate of the regularization boundaries in the suppressory $\varphi-\eta$ control plane.** Boundary function (cf. Eq. S31) encircling the region where chaos is suppressed in the $\varphi-\eta$ control plane for four values of the shape parameter: $m=0$ (dashed line), $m=0.717\simeq m_{\max}^{impulse}$ (solid line), $m=0.93$ (dotted line), and $m=0.96$ (thin solid line). System parameters as in Fig. S4.

text has the associated energy equation

$$\frac{dE}{dt}=-\delta\dot{x}^{2}+\gamma\dot{x}\sin\left(\omega t\right)-\beta\eta\dot{x}x^{3}f(t), \tag{S35}$$

where, for the sake of convenience, we introduced the shift $t\rightarrow t+T/4$, and hence $\varphi\rightarrow\varphi-\pi/2$, and where $E(t)\equiv(1/2)\,\dot{x}^{2}\left(t\right)+U\left[x\left(t\right)\right]$ $\left[U(x)\equiv-x^{2}/2+\beta x^{4}/4\right]$ is the energy function. Integration of Eq. S35 over *any* interval $[nT,nT+T/2]$, $n=0,1,2,...$, yields

$$\begin{aligned}E\left(nT+T/2\right)=E(nT)-\delta\int_{nT}^{nT+T/2}\dot{x}^{2}\left(t\right)dt-\beta\eta\int_{nT}^{nT+T/2}\dot{x}\left(t\right)x^{3}\left(t\right)f\left(t\right)dt\\+\gamma\int_{nT}^{nT+T/2}\dot{x}\left(t\right)\sin\left(\omega t\right)dt.\end{aligned} \tag{S36}$$

Now, if we consider fixing the parameters (δ,γ,β,T) for the Duffing oscillator to undergo chaotic behaviour at $\eta=0$, there always exists an $n=n^{*}$ such that the energy increment $\Delta E\equiv E\left(n^{*}T+T/2\right)-E(n^{*}T)$ is positive before chaotic escape from one of the two potential wells. Thus, after applying the first mean value theorem for integrals [5] together with well-known properties of the Jacobian elliptic functions [1] to the last two integrals on the right-hand side of Eq. S36,

$$\begin{aligned}E\left(n^{*}T+T/2\right)=E(n^{*}T)-\delta\int_{n^{*}T}^{n^{*}T+T/2}\dot{x}^{2}\left(t\right)dt+\frac{\gamma T}{\pi}\dot{x}\left(t^{*}\right)\\-\frac{\beta\eta T\dot{x}\left(t^{**}\right)x^{3}\left(t^{**}\right)}{2}F\left(\varphi,m\right),\end{aligned} \tag{S37}$$

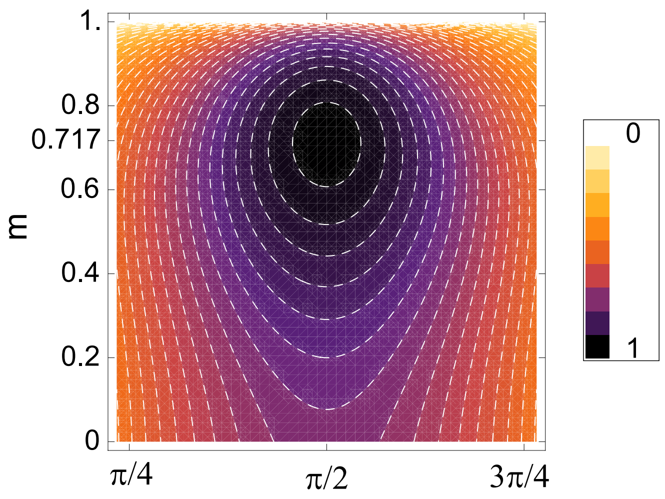

Figure S6: **Function $F(\varphi, m)$ describing the effect of the SE's impulse in the energy equation.** Contour plot of the function $F(\varphi, m)$ (see Eq. S38) versus the initial phase difference φ and the shape parameter m showing an absolute maximum at $\varphi = \varphi_{opt} = \pi/2, m = m_{\max}^{impulse} \simeq 0.717$. Note that the region around the value $\varphi = \varphi_{opt} = 3\pi/2$ is not shown because of the symmetry $F(\pi/2, m) = -F(3\pi/2, m) = N(m)/K(m)$ (cf. Eq. S40).

where $t^*, t^{**} \in [n^*T, n^*T + T/2]$ and

$$F(\varphi, m) \equiv \frac{\sqrt{1-m}N(m)}{K(m)} \operatorname{sd}\left[\frac{2K(m)\varphi}{\pi}\right], \tag{S38}$$

with $\operatorname{sd}(\cdot) \equiv \operatorname{sn}(\cdot; m) / \operatorname{dn}(\cdot; m)$ being the Jacobian elliptic function of parameter m, one has

$$\gamma T \dot{x}(t^*) / \pi > \delta \int_{n^*T}^{n^*T+T/2} \dot{x}^2(t)\, dt \tag{S39}$$

at $\eta = 0$ when the Duffing oscillator exhibits chaotic behaviour. It is straightforward to see that $F(\varphi, m)$ presents an absolute maximum (minimum) at $m = m_{\max}^{impulse} \simeq 0.717, \varphi = \pi/2$ ($m = m_{\max}^{impulse} \simeq 0.717, \varphi = 3\pi/2$). It is a 2π-periodic function in φ, and presents the noteworthy properties (see Fig. S6):

$$F(\pi/2, m) = -F(3\pi/2, m) = \frac{N(m)}{K(m)} = 2I(m), \tag{S40}$$

$$F(0, m) = F(\pi, m) = 0, \tag{S41}$$

$$\lim_{m\to 1} F(\pi/2, m) = \lim_{m\to 1} F(3\pi/2, m) = 0, \tag{S42}$$

$$\lim_{m\to 0} F(\pi/2, m) = -\lim_{m\to 0} F(3\pi/2, m) = \frac{2}{\pi}. \tag{S43}$$

In this situation, one lets the elliptic SE act on the Duffing oscillator while holding the remaining parameters constant. For sufficiently small values of $\eta > 0$, one expects that both the dissipation

work (the integral in Eq. S37) and $\dot{x}(t^{*})$ will approximately maintain their initial values (at $\eta = 0$) while the function $F(\varphi, m)$ will increase (decrease) from 0 (at $\varphi = 0, \pi$), so that, in some cases depending upon the remaining parameters and the sign of $\dot{x}(t^{**})\, x^3(t^{**})$, the energy increment just before the chaotic escape existing for $\eta = 0$, ΔE, could be sufficiently large and negative to suppress the initial chaotic state in the sense of leading the Duffing oscillator to the basin of a certain periodic attractor. Clearly, the probability of suppressing the initial chaotic state is maximal at $m = m_{\max}^{impulse} \simeq 0.717, \varphi = \pi/2$ $(\varphi = 3\pi/2)$ (i.e., when the impulse transmitted by the SE is maximum, cf. Eq. S40), which is in complete agreement with the foregoing MA-based predictions.

Remarkably, we can obtain an useful alternative estimate of the suppressory amplitude, η', by requiring that the sum of the two excitation terms in Eq. S37 be approximately cancelled:

$$\frac{\beta\eta'\dot{x}(t^{**})\, x^3(t^{**})}{2} F(\varphi, m) \approx \frac{\gamma}{\pi}\dot{x}(t^{*}). \tag{S44}$$

In such a case, the remaining integral in Eq. S37 (dissipation work) yields an energy decrease over time which suppresses the initial chaotic state, ultimately leading the Duffing oscillator to small-amplitude periodic oscillations around some of the two fixed points $\left(x = \pm\beta^{-1/2}, \dot{x} = 0\right)$ of the unperturbed Duffing oscillator $(\delta = \gamma = \eta = 0)$. From the properties of the function $F(\varphi, m)$ (cf. Eqs. S40-S43), one sees that the lower values of η' are obtained for $\varphi = \varphi_{opt} = \{\pi/2, 3\pi/2\}$, and hence an alternative estimate of the upper suppressory amplitude, $\eta'_{\max}$, reads

$$\frac{\eta'_{\max}(m)}{\eta'_{\max}(m=0)} \approx \frac{2/\pi}{|F(\pm\pi/2, m)|} \equiv \frac{2K(m)}{\pi N(m)} \equiv \left[\frac{I(m)}{I(m=0)}\right]^{-1}, \tag{S45}$$

which presents a single minimum at $m = m_{\max}^{impulse} \simeq 0.717$, while its behaviour, as a function of the shape parameter, is similar to that of the MA-based upper suppressory amplitude (cf. Eq. S23):

$$\frac{\eta_{\max}(m, T)}{\eta_{\max}(m=0, T)} = \left[\frac{a_0(m)}{a_0(m=0)} + \sum_{p=1}^{\infty} \frac{a_p(m) b_p(T)}{a_0(m=0) b_0(T)}\right]^{-1}. \tag{S46}$$

It is worth noticing that the approximate character of the suppressory condition given by Eq. S44 prevents us from ensuring that, even in certain cases corresponding to particular values of the initial conditions and system parameters, the SE can effectively lead the Duffing oscillator to some of the two fixed points $\left(x = \pm\beta^{-1/2}, \dot{x} = 0\right)$. Indeed, Eq. S37 tell us that any decrease of the Duffing oscillator's energy over half a period implies a subsequent decrease of the dissipation work over the next half a period, such that this decrease process continues until some of the mismatches of the (approximate) cancellation of the two excitation terms is sufficiently large to compensate

the dissipation work in the sense of yielding an increase of the energy, over a certain half a period, and a subsequent energy oscillation later. This means that the steady behaviour becomes a small-amplitude periodic oscillation around some of the fixed points from a certain instant $t = n^s T$, while the corresponding dissipation work is proportional to the action of the periodic orbit in the phase space:

$$\delta \int_{n^s T}^{n^s T+T/2} \dot{x}^2 (t)\, dt = \delta \int_{n^s T}^{n^s T+T/2} \dot{x} (t)\, dx = \delta \pi J, \tag{S47}$$

where $J \equiv \frac{1}{2\pi} \oint pdq$ is the action integral [6]. Alternatively, one can show the same behavior as follows. After linearizing Eq. (2) in the main text around $x = \pm\beta^{-1/2}$, one straightforwardly obtains the equation governing the linear stability of the two equilibria:

$$\ddot{z} + \omega_0^2 z = -\delta \dot{z} - \eta \left(3z \pm \beta^{-1/2}\right) f(t) + \gamma \cos(\omega t), \tag{S48}$$

where $\omega_0 \equiv \sqrt{2}$ and $z \equiv x \mp \beta^{-1/2}$, respectively. Equation S48 has the associated energy equation

$$\frac{dE_0}{dt} = -\delta \dot{z}^2 + \gamma \dot{z} \sin(\omega t) - \eta \left(3z \pm \beta^{-1/2}\right) z f(t), \tag{S49}$$

where we introduced the shift $t \to t+T/4$, and hence $\varphi \to \varphi-\pi/2$, and where $E_0(t) \equiv (1/2)\, \dot{z}^2 (t) + U_0 [z(t)]$ $\left[U_0(z) \equiv \omega_0^2 z^2/2\right]$ is the energy function of the linearized system. Integration of Eq. S49 over *any* interval $[nT, nT + T/2]$, $n = 0, 1, 2, ...$, yields

$$\begin{aligned} E_0 (nT + T/2) = E_0(nT) &- \delta \int_{nT}^{nT+T/2} \dot{z}^2 (t)\, dt + \gamma \int_{nT}^{nT+T/2} \dot{z}(t) \sin(\omega t)\, dt \\ &- \eta \int_{nT}^{nT+T/2} \dot{z}(t) \left[3z(t) \pm \beta^{-1/2}\right] f(t)\, dt. \end{aligned} \tag{S50}$$

After applying the first mean value theorem for integrals together with well-known properties of the Jacobian elliptic functions to the last two integrals on the right-hand side of Eq. S50, one obtains

$$\begin{aligned} E_0 (nT + T/2) = E_0(nT) &- \delta \int_{nT}^{nT+T/2} \dot{z}^2 (t)\, dt + \frac{\gamma T}{\pi} \dot{z}(t') \\ &- \frac{\eta T \dot{z}(t'') \left[3z(t'') \pm \beta^{-1/2}\right]}{2} F(\varphi, m), \end{aligned} \tag{S51}$$

where $t', t'' \in [nT, nT + T/2]$. Note that the suppressory condition given by Eq. S44 implies the approximate cancellation of the sum of the two excitation terms in Eq. S51, and hence the same reasoning applied above to the general energy E can now be directly applied to the small-amplitude energy E_0 (compare Eqs. S37 and S51), thus allowing us to conclude that the regularized small-amplitude periodic oscillations around any of the fixed points $\left(x = \pm\beta^{-1/2}, \dot{x} = 0\right)$ are linearly stable attractors.

II. NUMERICAL METHODS

In our numerical simulations, we studied the purely deterministic case as well as the robustness of the impulse-induced chaos-control scenario against the presence of additive noise in the Duffing equation:

$$\ddot{x} = x - \beta \left[1 + \eta f(t)\right] x^3 - \delta \dot{x} + \gamma \cos(\omega t) + \sqrt{\sigma}\xi(t), \tag{S52}$$

where $\xi(t)$ is a Gaussian white noise with zero mean and $\langle \xi(t) \xi(t+s) \rangle = \delta(s)$, and $\sigma = 2k_b T^*$ with k_b and T^* being the Boltzmann constant and temperature, respectively. For the sake of completeness, we computed three types of complementary diagrams.

On the one hand, we compare the theoretical predictions obtained from MA with the Lyapunov exponent (LE) calculations for Eq. S52. In this regard, it is worth recalling that, even in the case of small values of γ, δ and η, one cannot expect too good a quantitative agreement between these two kinds of approaches because MA is a perturbative technique generally related to transient chaos, while LE provides information solely concerning steady responses. We computed the LEs using a version of the algorithm introduced in [7], with integration typically up to 10^4 drive cycles for each fixed set of parameters. In the absence of the SE ($\eta = 0$), the Eq. S52 with $\sigma = 0, \delta = 0.25, \gamma = 0.29, \beta = 1, \omega = 1$ exhibits a strange chaotic attractor with a maximal LE $\lambda^+(\eta = 0) = 0.025$ bits/s. To construct the LE diagrams we followed two steps. First, the maximal LE was calculated for each point on a $N \times N$ grid with phase difference φ and amplitude η along the horizontal and vertical axes. Second, a diagram was constructed by only plotting points on the grid according to a colour code. Periodic and chaotic solutions were detected. Since we solely consider the case of the main resonance ($T = 2\pi/\omega$) between the two involved excitations, quasiperiodic solutions were not detected, as expected [8].

On the other hand, we computed period-distribution and isospike diagrams [9] to obtain detailed information regarding the periodicity order of the regularized solutions as well as useful information regarding the complexity of their waveforms in the $\varphi - \eta$ control plane. Isospike diagrams are based on computing the number of local maxima per period for the periodic solutions after a sufficiently long transient for each point on a $N \times N$ grid with phase difference φ and amplitude η along the horizontal and vertical axes. To this end, after the first 10^4 drive cycles, we continued the integration for 200 additional drive cycles recording up to 800 extrema (maxima and minima) of the variable of interest and checking whether pulses repeated or not.

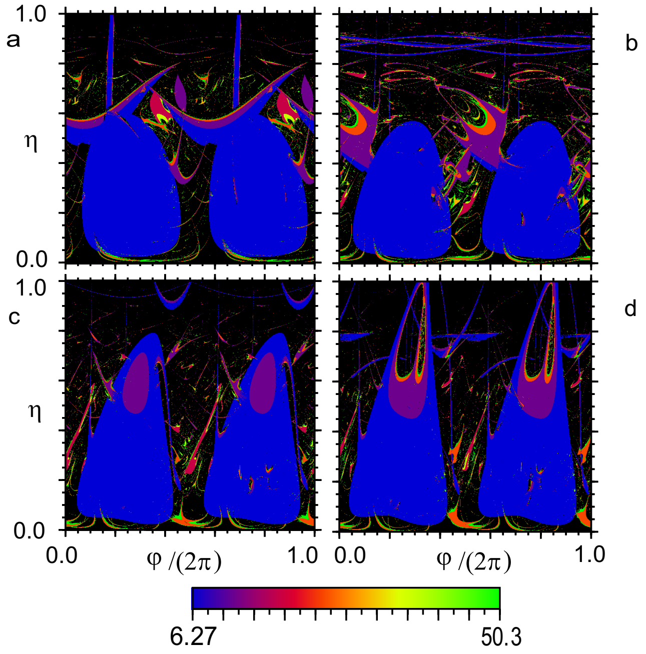

Figure S7: **Period-distribution diagrams.** Numerically calculated regularization regions according to the period of their periodic solutions and chaotic regions (black) in the $\varphi - \eta$ control plane for four values of the shape parameter: (a) $m = 0$, (b) $m = 0.717 \simeq m_{\max}^{impulse}$ (i.e., the m value at which the SE's impulse is maximum), (c) $m = 0.9$, and (d) $m = 0.95$. Fixed parameters: $\delta = 0.25, \gamma = 0.29, \beta = 1, \omega = 1$.

In isospike diagrams, black is used to represent chaos; i.e., lack of numerically detected periodicity. To represent maxima, we used a palette of 17 colors. Patterns with more than 17 maxima are plotted by recycling the 17 basic colors modulo 17. Period-distribution diagrams are based on computing the period of periodic solutions after a sufficiently long transient (10^4 drive cycles) for each point on a $N \times N$ grid with phase difference φ and amplitude η along the horizontal and vertical axes. In period-distribution diagrams we used a colour code to detect periodic solutions with periods between T (period-1 solution) and $8T$ (period-8 solution). In period-distribution diagrams, black is used to represent chaos; i.e., lack of numerically detected periodicity.

We studied the evolution of the regularization regions in the $\varphi - \eta$ control plane as the impulse transmitted by the SE is changed from its value at $m = 0$ to its value at an m value very close to 1 by computing LE, isospike, and period-distribution diagrams. For the purely deterministic case, the results are respectively shown in Figs. 4 and 5 of the main text and Fig. S7, while Fig. S8 shows, for the same set of fixed parameters, four illustrative LE diagrams for the Duffing oscillator in the presence of noise ($\sigma > 0$). Although the presence of noise gives systematically rise to a decrease, or even a complete elimination, of secondary and minor islands of regularization in the

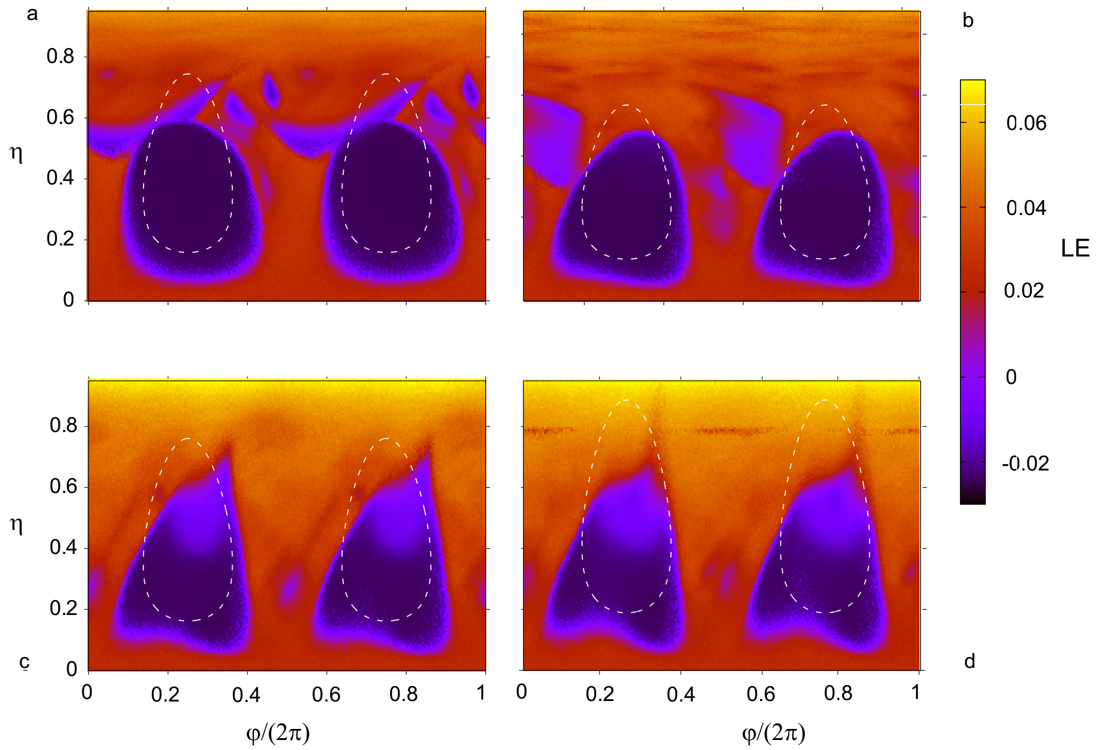

Figure S8: **Robustness of the impulse-induced chaos-control scenario against the presence of noise.** LE diagrams in the $\varphi - \eta$ control plane in the presence of noise for four values of the shape parameter: **a**, $m = 0$; **b**, $m = 0.717 \simeq m_{\max}^{impulse}$; **c**, $m = 0.9$; **d**, $m = 0.95$. The white contours indicate the respective predicted boundary functions for the purely deterministic case (cf. Eq. S31) which are symmetric with respect to the optimal suppressory values of the initial phase difference. Noise strength: $\sigma = 0.006$, and the remaining parameters as in Fig. S4.

$\varphi - \eta$ control plane (see Fig. S9), a comparison between the purely deterministic case ($\sigma = 0$) and the noisy case ($\sigma > 0$) for the same values of the shape parameter (compare Fig. 4 in the main text with Fig. S8) indicates that the impulse-induced chaos-control scenario is robust against the presence of moderate noise.

III. EXPERIMENTAL SETUP

The experimental setup used in our analog implementation of the damped driven Duffing oscillator (Eq. 2 in the main text) is shown in Fig. S10.

[1] Armitage, J. V & Eberlein, W. F. *Elliptic Functions* (Cambridge University Press, 2006).

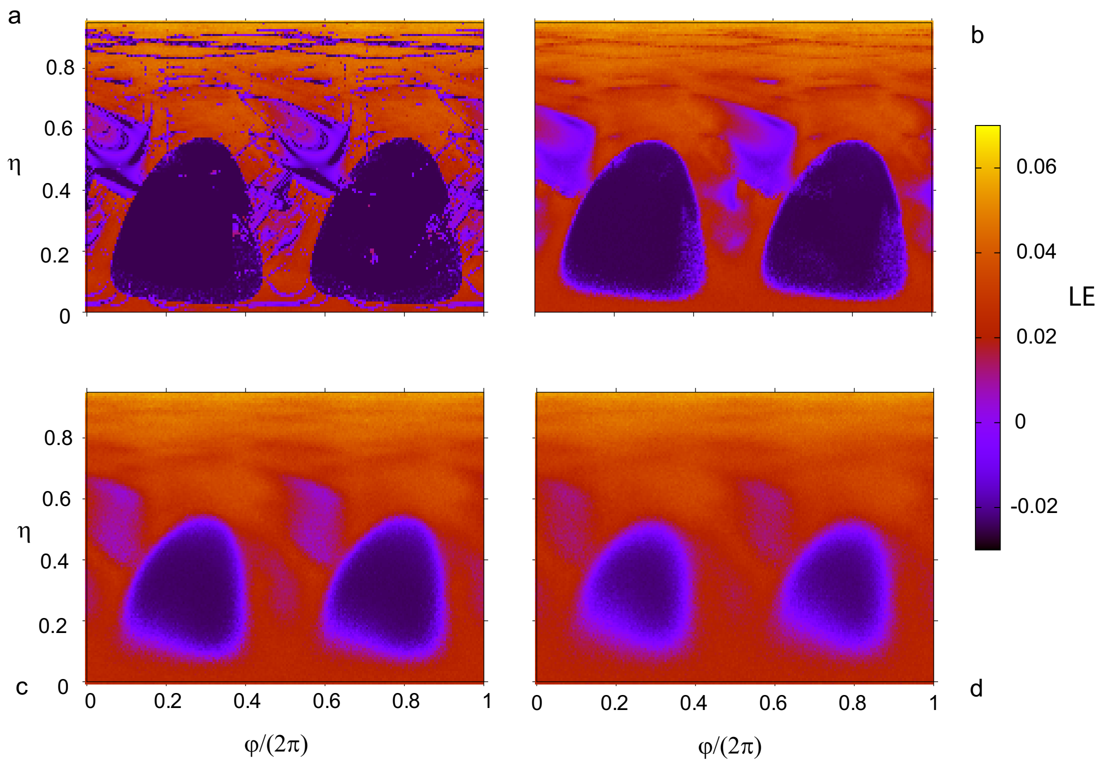

Figure S9: **Robustness of the maximal islands of regularization against increasing noise.** LE diagrams in the $\varphi-\eta$ control plane for four values of the noise strength: **a**, $\sigma = 0$ (purely deterministic case); **b**, $\sigma = 0.002$; **c**, $\sigma = 0.018$; **d**, $\sigma = 0.038$. Shape parameter: $m = 0.717 \simeq m_{\max}^{impulse}$, and the remaining parameters as in Fig. S4.

[2] Melnikov, V. K. On the stability of the center for time periodic perturbations. *Trans. Mosc. Math. Soc.* **12**, 1-57 (1963).

[3] Guckenheimer, J. & and Holmes, P. *Nonlinear Oscillations, Dynamical Systems, and Bifurcations of Vector Fields* (Springer-Verlag, 1983).

[4] Chacón, R. *Control of Homoclinic Chaos by Weak Periodic Perturbations* (World Scientific, 2005).

[5] Gradshteyn, I. S. & Ryzhik, I. M. *Table of Integrals, Series, and Products* (Academic Press, 1980).

[6] Lichtenberg, A. J. & Lieberman, M. A. *Regular and Stochastic Motion* (Springer-Verlag, 1983).

[7] Wolf, A., Swift, J. B., Swinney, H. L. & Vastano, J. A. Determining Lyapunov exponents from a time series. Physica D **16**, 285-317 (1985).

[8] Chacón, R & Martínez García-Hoz, A Route to chaos via strange non-chaotic attractors by reshaping periodic excitations. Europhys. Lett. **57**, 7-13 (2002).

[9] Freire, J. G. & Gallas, J. A. C. Stern-Brocot trees in the periodicity of mixed-mode oscillations. *Phys. Chem. Chem. Phys.* **13**, 12191-12198 (2011).

[10] Meucci, R., Euzzor, S., Zambrano, S., Pugliese, E., Francini, F. & Arecchi, F. T. Energy constraints in pulsed phase control of chaos. *Phys. Lett. A* **381**, 82-86 (2017).

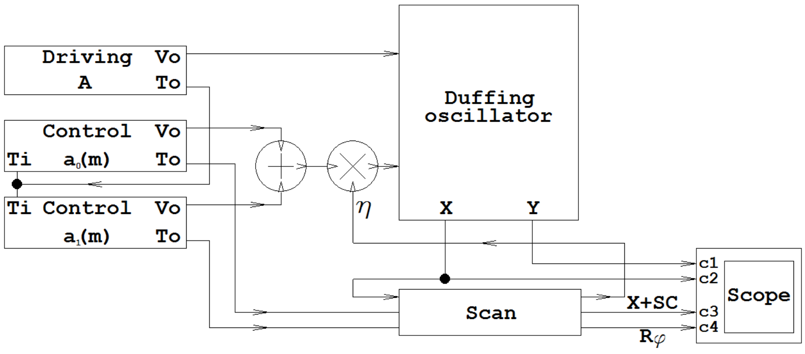

Figure S10: **Scheme of the Duffing's oscillator circuit.** Blocks diagram of a damped two-well Duffing oscillator driven by a sinusoidal chaos-inducing signal and subjected to an elliptic suppressory signal in the form of a parametric perturbation of the cubic term. It includes the damped Duffing oscillator block with outputs x and y, a driving block which generates the sinusoidal chaos-inducing signal, while the control blocks generate the two-harmonics approximation of the elliptic suppressory signal. The scan block performs an automatic scanning of the initial phase difference φ and the suppressory amplitude η through the ramp signal R_ϕ and the staircase signal SC.

[11] Meucci, R., Euzzor, S., Pugliese, E., Zambrano, S., Gallas, M. R. & Gallas, J. A. C. Optimal phase-control strategy for damped-driven Duffing oscillators. *Phys. Rev. Lett.* **116**, 044101 (2016).
